# Switchable genetic oscillator operating in quasi-stable mode

**DOI:** 10.1098/rsif.2009.0487

**Published:** 2010-01-22

**Authors:** Natalja Strelkowa, Mauricio Barahona

**Affiliations:** Department of Bioengineering and Institute for Mathematical Sciences, Imperial College London, South Kensington Campus, London SW7 2AZ, UK

**Keywords:** synthetic biology, oscillatory gene networks, generalized repressilator, travelling waves, stochastic dynamics

## Abstract

Ring topologies of repressing genes have qualitatively different long-term dynamics if the number of genes is odd (they oscillate) or even (they exhibit bistability). However, these attractors may not fully explain the observed behaviour in transient and stochastic environments such as the cell. We show here that even repressilators possess quasi-stable, travelling wave periodic solutions that are reachable, long-lived and robust to parameter changes. These solutions underlie the sustained oscillations observed in even rings in the stochastic regime, even if these circuits are expected to behave as switches. The existence of such solutions can also be exploited for control purposes: operation of the system around the quasi-stable orbit allows us to turn on and off the oscillations reliably and on demand. We illustrate these ideas with a simple protocol based on optical interference that can induce oscillations robustly both in the stochastic and deterministic regimes.

## 1. Introduction

Recent experimental advances in cellular and molecular biology have made it possible to engineer intricate gene regulatory circuits (Andrianantoandro *et al.* 2006). Inspired in many cases by electronic elements, simple gene networks have been designed to perform reproducible, low-level functions. Some classic examples include the toggle switch (Gardner *et al.* 2000), the genetic ring oscillator known as the repressilator (Elowitz & Leibler 2000) or a circuit that can exhibit both oscillatory and switching behaviour through the alteration of biochemical interactions (Atkinson *et al.* 2003). Such simple circuits could be potentially interconnected and built up to form more elaborate ‘biological devices’ with large numbers of components. This trend is facilitated by simulation software containing large numbers of genes (Marchisio & Stelling 2008) as well as libraries of composable biological parts for experimental realization (MIT 2009). Simple synthetic modules can also be integrated into the complex machinery of the cell, as in the oscillator recently implemented in a mammalian cell (Tigges *et al.* 2009), or interfaced with cellular pathways to induce particular responses, as in the construct where the toggle switch was connected to the SOS pathway to induce DNA protection mechanisms in *Escherichia coli* when exposed to ultraviolet (UV) light (Kobayashi *et al.* 2004). Similar principles have been exploited in the rational design of internal negative feedback operated in conjunction with external arabinose-driven positive feedback to produce cell-synchronized oscillations (Stricker *et al.* 2008).

The central role played by oscillations in cellular function has made oscillatory circuits a primary target for the analysis and design of synthetic networks. A particular area of interest is the elucidation of strategies leading to robust timing and sequential activation in the cell. For instance, key stages in developmental biology and in cell differentiation may be controlled by so-called master regulators—a small set of transcription factors sequentially activating and driving several other genes with accurate timing (Borneman *et al.* 2006; Liu *et al.* 2007; Bondue *et al.* 2008). In addition, studies of both natural (Megerle *et al.* 2008; Spencer *et al.* 2009) and engineered circuits (Glick 1995) indicate that the correct timing and order of gene activation is a key characteristic of balanced, optimal cell function, as it reduces the metabolic burdening that ensues from the continuous presence of heterologous substances (A. Glieder 2009, personal communication).

**Figure 1.**
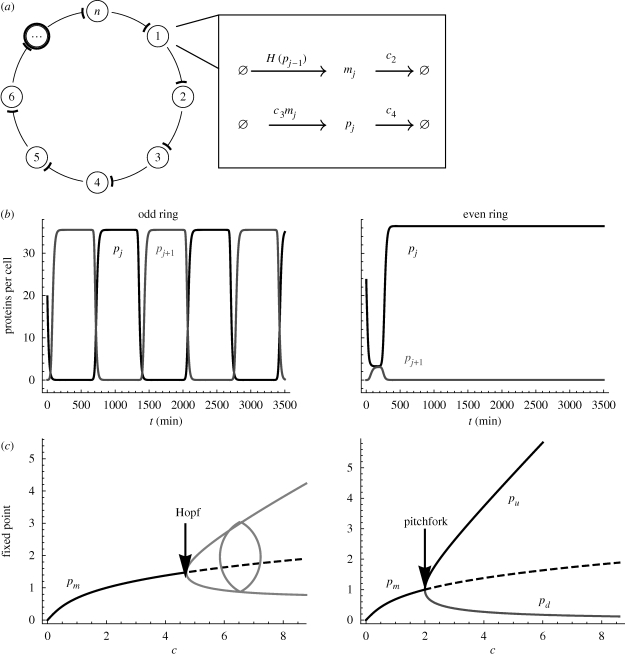
Attractors of the generalized repressilator model. (*a*) Topology of the generalized repressilator: *n* genes in a cycle where each gene is repressed by the protein product of the preceding gene. Also shown is the reaction scheme underlying the dynamical system (1) with production and degradation terms for the mRNA (*m*_*j*_) and protein (*p*_*j*_) of each gene. The repression of the production of mRNA is modelled by a Hill-type term *H*(*p*_*j*−1_). (*b*) Typical time traces of the long-term deterministic dynamics of an odd ring and an even ring above the bifurcation point, *c* = 2: odd rings converge to a globally attracting periodic solution while even rings converge to fixed points. The time traces shown correspond to *n* = 23 and *n* = 22. (*c*) Stability of the fixed points of the system as a function of the bifurcation parameter *c*. Even rings undergo a pitchfork bifurcation at *c* = 2, leading to the emergence of two stable fixed points. Odd rings undergo a Hopf bifurcation leading to the emergence of a limit cycle. The critical parameter for the Hopf bifurcation depends on *n* but tends to *c* = 2 as *n* grows (see the electronic supplementary material).

In this paper, we consider the dynamics and control of noisy genetic oscillatory circuits in quasi-stable mode operation. We exemplify our results with one of the simplest and most widely studied synthetic networks: the *n*-gene ring repressilator (figure 1*a*). Some natural networks of master regulators (Borneman *et al.* 2006) contain such ring structures as subnetworks, making the exploration of their dynamic behaviour relevant for both naturally occurring and synthetic systems. The underlying idea is well-known: when observing the dynamics of systems operating in highly variable environments, such as the cell, it might not be enough to characterize only the long-term attractors of the system since unstable solutions can play a significant role. For instance, quasi-stable transients might be so long-lived as to be the most significant feature of the observed noisy dynamics (Trefethen & Embree 2005). Moreover, the presence of noise in nonlinear systems may induce non-stationary dynamics in systems with only fixed point attractors in the deterministic setting (Süel *et al.* 2006) or, conversely, noise may act as a stabilizer of unstable deterministic states (Turcotte *et al.* 2008).

In the generalized repressilator, results due to Smith (1987) and Müller *et al.* (2006) show that rings with an even number of genes (e.g. the toggle switch (Gardner *et al.* 2000) with *n* = 2) exhibit multistability and hence behave like switches in the stochastic regime. On the other hand, rings with an odd number of genes (like the standard repressilator (Elowitz & Leibler 2000) with *n* = 3) have a globally attracting limit cycle and are therefore oscillators both in the stochastic and deterministic regimes. However, here we show that generalized repressilators possess an intricate structure of unstable periodic orbits that play an important part in their observable noisy and transient dynamics. In particular, even rings have a quasi-stable limit cycle which, although unstable in terms of linear Floquet stability analysis, has only one unstable direction with a very slow escape rate. This means that trajectories are attracted to the limit cycle from all directions but one, hence leading to long-lived, inducible periodic transients in the deterministic setting and to sustained oscillations in the stochastic system. These effects become more pronounced as the number of genes grows. Therefore, the finite-time, observable noisy dynamics of an even repressilator ring is not necessarily static (switch-like) but rather exhibits oscillatory characteristics.

In addition to their effect on the observable dynamics, quasi-stable oscillatory modes can be used as operating points to control the system around them. The advantage of such a scheme is that the oscillations can be switched on and off, unlike the limit cycle attracting behaviour of odd repressilators. Operation around unstable modes, usually illustrated with an example of the inverted pendulum (Franklin *et al.* 1993), is a classic scenario in control theory for enhanced controllability and speed of response. It has a long and successful history of applications in fluid flow control (Ahuja & Rowley 2009) and in the steering of jet aircraft (McRuer & Graham 2004). Here, we illustrate the application of this concept to gene networks with a simple protocol of controlled interference based on an optical mechanism for readout and induction of gene expression. The current concept is based on an alternative mechanism to the chemical intervention proposed in Atkinson *et al.* (2003) to produce switchable oscillations. Our simulations show that even repressilator rings in quasi-stable operation can behave as a robust and on-demand switchable oscillator in which genes become upregulated periodically in an ordered sequence according to a travelling wave solution. This switchability, which is robust at both high and low copy numbers, could be used for synthetic biological applications such as accurately timed interference with naturally occurring networks.

## 2. Theory

### 2.1. Model equations and stability of fixed points

The generalized repressilator consists of a ring of *n* genes in which transcription of each gene is repressed by the product of the preceding gene (figure 1*a*). A deterministic model of this circuit is given by the following set of ordinary differential equations:
2.1
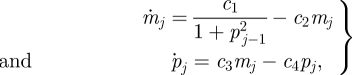

where *p*_*j*_ and *m*_*j*_ describe protein and mRNA concentrations for each gene, respectively (Elowitz & Leibler 2000). Here, *j* = 1, … ,*n* with the periodic boundary condition *p*_0_ = *p*_*n*_, and *c*_1_ (*c*_3_) is the creation rate and *c*_2_ (*c*_4_) is the degradation rate for the mRNAs (proteins). The production of mRNA is modelled as a source term that depends nonlinearly on the concentration of the inhibitor protein. Proteins are assumed to be produced at a rate linearly dependent on the amount of the corresponding mRNA. The degradation of mRNA and proteins is assumed to be linearly proportional to their current amount. The toggle switch (Gardner *et al.* 2000) and the repressilator oscillator (Elowitz & Leibler 2000), which have both been implemented in *E. coli*, are special cases with *n* = 2 and *n* = 3, respectively. Based on analytical results on monotone systems (i.e. systems in which partial derivatives do not change sign) owing to Smith (1987) and Müller *et al.* (2006), the stability analysis of this circuit reveals a fundamental difference between rings with odd and even numbers of genes. We briefly sketch some of the main results, which are also summarized in figure 1*b*,*c*.

The stability analysis characterizes the long-term dynamic behaviours of the deterministic system. An example of such behaviour is given by the fixed points of the system, i.e. the states in which the dynamics is stationary. The variation of a parameter can produce a change in the stability or the existence of fixed points or other attractors. This is called a bifurcation and it leads to qualitative changes in the long-term behaviour of the system. One can find the parameter values at which bifurcations are produced by performing a bifurcation analysis, which can be carried out analytically (in some simple cases) or numerically with the aid of continuation software packages such as Auto (Doedel 2007), which can also track the stability of periodic solutions.

In our system equation (2.1), the fixed points, where all derivatives are zero, are found from the condition
2.2
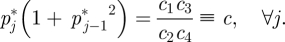



The parameter *c* defined in equation (2.2) will play the role of the bifurcation parameter for even rings. A positive and real solution is obtained if all proteins have the same concentration: 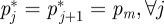 and

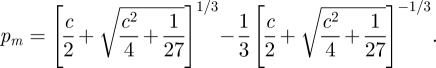



This solution, which exists as long as *c* is positive, is stable for small *c* and becomes unstable for larger values of *c* in both odd and even rings.

In the case of even rings, a pitchfork bifurcation takes place at *c* = 2 for all *n* (figure 1*c*). The two additional stable fixed points arising at that value of the parameter correspond to 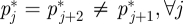, which gives
2.3
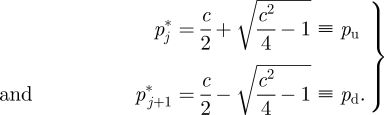



Note that *p*_u_ → *c* − 1/*c* and *p*_d_ → 1/*c* for large *c*. The new fixed points of system (2.1) correspond to two distinct dimerized states: one in which genes with odd indices are upregulated (*p*_u_), while genes with even indices are downregulated (*p*_d_); and another symmetric state where the genes with odd and even indices exchange their patterns of regulation. These solutions are equivalent to tiling the ring with *n*/2 repeated copies of the up–down solution of the two-gene ring. Their structure is similar to that of other dimerized degenerate solutions in classic models of conjugated polymers and spin chains (Soos 2007). Therefore, after the bifurcation, the system is bistable, i.e. it behaves like a switch in the presence of noise.

In the case of odd rings (figure 1*b*), *p*_*m*_ becomes unstable following a bifurcation that occurs at a value *c*(*n*) that approaches 2 as *n* grows. However, in this case the bifurcation is Hopf: no additional fixed points appear but rather the bifurcation signals the emergence of a periodic solution. Smith (1987) proved that in monotone systems such as the repressilator, the periodic solution that emerges is a globally attracting stable limit cycle. Therefore, odd rings behave as stable oscillators following the Hopf bifurcation (HB).

### 2.2. Floquet theory and unstable periodic orbits

The stability analysis presented above does not provide information about unstable periodic solutions. Although, in principle, unstable periodic orbits are not relevant for the long-term deterministic dynamics, they can be key to the observed dynamics, especially if the orbits involve slow time scales. Such long-lived oscillations can appear as transients in deterministic simulations and are likely to be observed in the corresponding stochastic simulations. In fact, it was in numerical simulations that we first noticed the relevance of these modes in even repressilator rings.

Floquet theory can be used to find periodic solutions and quantify their linear stability in terms of their Poincaré map, i.e. the crossings of the orbit with a (hyper)plane in phase space. Under this analysis, a periodic solution (a closed orbit) becomes a fixed point of the Poincaré map and its stability is reformulated as the linear stability of this fixed point. The eigenvalues of the Poincaré map linearized around the fixed point constitute the Floquet multipliers. They indicate how an infinitesimal perturbation around the orbit decays or grows (exponentially). The periodic solution is linearly stable if all the Floquet multipliers have magnitudes smaller than unity (see the references and details on Floquet theory in the electronic supplementary material). In some cases, a few (possibly only one) Floquet multipliers will be slightly larger than one. We will then have ‘quasi-stable’ periodic solutions in that it takes a long time to diverge away from them. Quasi-stability in this sense is a local property. To assess if these solutions will be reachable (and therefore observable in the dynamics), one needs to employ global techniques, e.g. sampling the space of initial conditions. However, Floquet analysis provides an indication of the possibility of observable, yet unstable, periodic solutions. If a periodic orbit has a small number of very weakly unstable directions, it is likely that it could be observed as long-lived periodic transients in the deterministic system and that it could also play a role in the stochastic dynamics. Moreover, such quasi-stable orbits are good targets for a control mechanism that can make oscillations switchable, as shown below.

## 3. Methods

### 3.1. Numerical simulations and analysis of the dynamics

The deterministic system of ordinary differential equations (2.1) was solved numerically with an adaptive fourth-order Runge–Kutta integrator (Press *et al.* 1992), in which the step-size automatically adapts to meet the required accuracy *ϵ*. We have checked that the inducibility, reachability and transient times of the quasi-stable oscillations are not affected by the accuracy of the integrator by using the Runge–Kutta integrator with accuracies *ϵ* between 10^−2^ and 10^−8^ (see the details in the electronic supplementary material). In addition, the observability of the unstable orbits was confirmed by using a nonlinear integrator (Zi *et al.* 2008).

The bifurcation analysis and the calculation of the Floquet multipliers of the unstable periodic orbits were carried out with the numerical continuation software Auto (Doedel 2007; see details in the electronic supplementary material).

Stochastic simulations of the generalized repressilator were performed using the classical Gillespie algorithm (Gillespie 1977). Random numbers and quasi-random numbers for numerical simulations were generated with the GSL Scientific Library (Galassi *et al.* 2009).

### 3.2. Global robustness analysis and control aspects of quasi-stable oscillations

As part of our numerical evaluation of the generalized repressilator, we have developed a method to carry out a robustness and reachability analysis of its quasi-stable oscillations. This was necessary because available global robustness tools (Zi *et al.* 2008) quantify changes in fixed points induced by parameter variations and are therefore not directly applicable to oscillations. In order to evaluate the global robustness and inducibility of the quasi-stable oscillations, we attempt to induce sustained oscillations with a predetermined intervention and quantify changes in the observed response when the model parameters are varied. The method defines an operating point in parameter space (the reference set c*_j_), based on biologically appropriate estimates, and a hypercube around it to account for biological variability, temperature gradients and other noise. We then sample parameter sets from the hypercube using reverse halton sequences (Halton 1960), quasi-random sequences that have been shown to converge faster than standard Monte Carlo sampling for high-dimensional spaces (Vandewoestyne & Cools 2006). For each sampled parameter set, we attempt to induce oscillations with the STOP–KICK scenario described below and record if the system evolves towards sustained oscillations. If oscillations are observed, we numerically calculate the period of the oscillation and the change in shape (see details in the electronic supplementary material). Characterizing the change in shape is essential to establish that the oscillation remains detectable and functionally recognizable in the biological system. Note that here we are only concerned with global robustness of the reachability of the solution. A modification of the same algorithm could be used to study the parameter combinations that contribute most strongly to the sensitivity of the network, a question relevant for the experimental tuning of the system that is not addressed here.

## 4. Results

### 4.1. Stable and quasi-stable oscillations in the generalized repressilator

**Figure 2.**
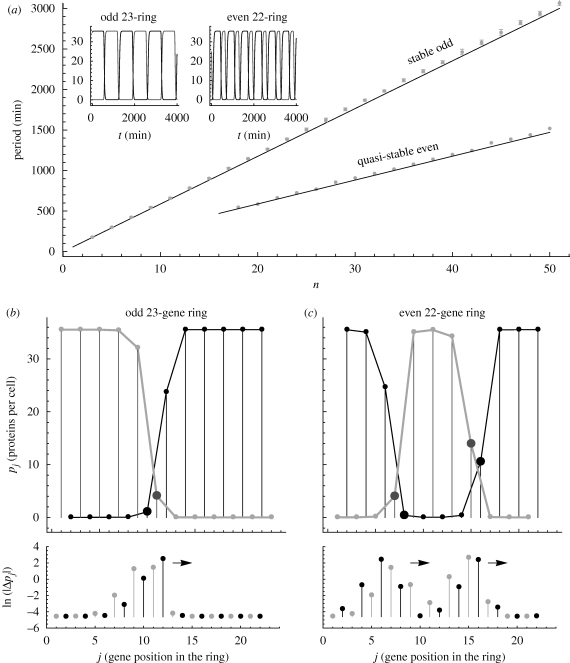
Periodic solutions and travelling waves in the generalized repressilator model. (*a*) The period of the limit cycle of the deterministic model of odd rings (solid line) increases linearly with the length of the ring. Simulations of the stochastic version of this system using the Gillespie algorithm show that the period (shown as circles) follows the same trend, although they are slightly larger. The period of the quasi-stable solutions found in even rings (deterministic and stochastic) increases linearly with the number of genes but with a slope that is half that of the odd rings. The inset shows representative time traces of the periodic solutions in odd rings (stable) and in even rings (quasi-stable). (*b*) Time snapshot of the spatial distribution of the concentrations of two successive protein concentrations for the periodic solution in the odd ring with *n* = 23. The solution has a travelling wave structure with a kink-like perturbation propagating around the ring, indicated by the arrow in the bottom figure. The bottom figure represents the minimum distance |*Δp*_*j*_| = min(|*p*_u_ − *p*_*j*_|, |*p*_d_ − *p*_*j*_|) between the travelling wave solution and the dimerized solution with an alternating pattern of protein expression given by *p*_u_ and *p*_d_. The distance becomes large around the kink in the travelling wave solution. (*c*) Same as (*b*) for the quasi-stable periodic solution of the even ring with *n* = 22. In this case, the travelling wave solution has two kinks that propagate around the ring, as indicated by the arrows.

As pointed out in the §2, odd repressilator rings are globally attracted to stable limit cycle oscillations for *c* > 2. Numerical simulations show that the period of these solutions increases linearly with the number of genes in the ring (figure 2*a*). The stability analysis also shows that, in contrast, even rings only support fixed points as stable solutions. However, direct dynamical simulations of even repressilator rings reveal the existence of long-lived periodic solutions, which are easily reachable, as checked by extensive sampling (not shown) of the space of initial conditions. The period of these oscillations also increases linearly with the number of genes, albeit with a slope that is approximately half of that in odd rings (figure 2*a*).

These numerical observations do not pose a contradiction with the stability analysis above: the observed oscillations in even rings are periodic solutions yet unstable. Unstable solutions can be studied using the numerical bifurcation detection software Auto (Doedel 2007), a continuation package that does not rely on dynamical simulations (see the electronic supplementary material). We have used Auto to find bifurcations in the biologically relevant range of the parameter *c* and to assess the linear stability of fixed points and periodic solutions—the latter through Floquet analysis.

**Figure 3.**
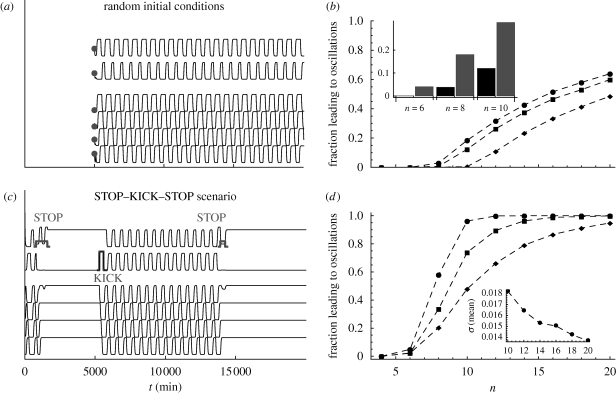
Induction of oscillations in even rings in the deterministic regime. (*a*) Quasi-stable periodic solutions in even repressilator rings can be observed starting from random initial conditions. The dynamical trajectories shown here correspond to a ring with *n* = 18 and parameters *c*_1_ = 2.6, *c*_2_ = 0.12, *c*_3_ = 0.2, *c*_4_ = 0.06. (*b*) Percentage of initial conditions leading to 5 (filled circles), 10 (filled squares) and 50 (filled diamonds) oscillations. The percentages are obtained from a sampling of 10^4^ initial conditions. The inset shows the percentage of initial conditions leading to oscillations increases in the stochastic regime (see the electronic supplementary material). (*c*) The quasi-stable oscillations can be induced reliably with a simple sequence of signals. First, apply a STOP signal to gene *j* to force the system to approach a fixed point solution. Second, apply a KICK signal to gene *j* + 1 to drive the ring into oscillation. The oscillation can be terminated at will by applying another STOP signal. The signals can be implemented via on-demand UV or red light gene transcription activation (Shimizu-Sato *et al.* 2002). This STOP–KICK–STOP protocol is shown here for the same ring as in (*a*). (*d*) Global robustness of the inducibility of the quasi-stable oscillations. The STOP–KICK scenario is applied to 10^4^ random combinations of parameters *c*_*j*_ for each even ring of length *n* and we record the proportion of parameter sets that lead to five oscillations. The parameters are sampled with reverse Halton sequences from a hypercube with 5 per cent (filled circles), 10 per cent (filled squares) and 20 per cent (filled diamonds) variation around the reference set. Quasi-stationary oscillations are robustly induced for *n* ≥ 10 (see the electronic supplementary material for results concerning the production of 10 oscillations). Small rings can be kept in the oscillating state applying repeated interventions in a simple control protocol, as seen in figure 4. The inset shows the oscillations are robust in shape (see the electronic supplementary material) and in period with respect to changes in the parameters. The relative variability (coefficient of variation) of the period of the induced oscillations is small and decreases with the length of the ring.

The result for even rings is presented in table 1. In agreement with the analytical results, a pitchfork bifurcation is found numerically as a branching point at *c* = 2, above which HBs leading to the appearance of unstable periodic solutions are detected in all rings with more than four genes. The Floquet stability analysis indicates that the first unstable periodic orbit to emerge has only one unstable direction, regardless of the number of genes. The only positive Floquet multiplier, which indicates how fast the trajectory diverges away from the orbit, is small and decreases as the length of the ring increases. This is the signature of quasi-stability: if this periodic orbit is reached, it will be long-lived. We have also checked that this solution is reachable through numerical sampling of the space of initial conditions (figure 3*a*,*b* and electronic supplementary material). Such reachable quasi-stable modes significantly affect the observed transient dynamics and also play a central role in stochastic dynamics, where unstable solutions are explored under the effect of noise. Both these conditions are relevant for dynamics of genetic circuits inside the cell.

**Figure 4.**
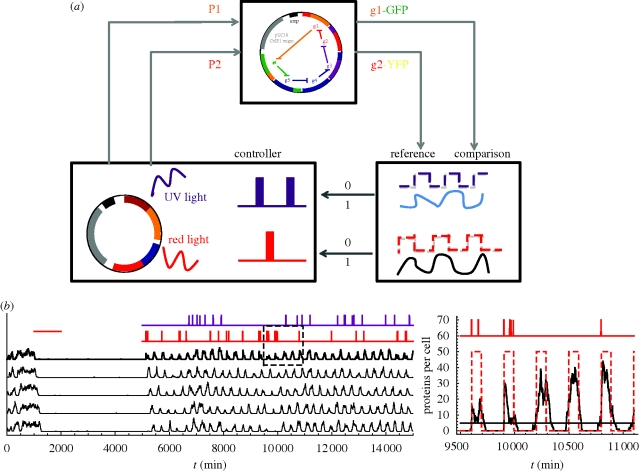
Stochastic oscillations in even rings and readout-based control. (*a*) Illustration of the readout-based control scheme for a ring of six genes. Two proteins of the ring are read out with fluorescent tags. This readout is then compared with a reference defined according to the oscillating behaviour of the ring, with similar period and a shift between consecutive genes. The reference comparison is threshold-based and leads to an ON–OFF (1–0) control for the KICK signals. These can be implemented with light-responsive gene promoters. In the numerical simulations shown in (*b*), the KICK signals are indicated with the red markings in the upper panels. (*b*) A simple readout-based control reliably switches on the oscillations, sustains them and switches them off. The control mechanism functions by monitoring two successive proteins in the ring. Whenever each of them falls below a threshold, a KICK signal for the corresponding protein is given. These threshold-based KICK signals are indicated with red and magenta markings in the upper panels. The oscillation can be terminated with a STOP signal as in the deterministic state. The optical readout can be based on GFP or YFP protein labelling, while the response can be implemented with on-demand UV or red light that enhances the production of the corresponding mRNAs (Shimizu-Sato *et al.* 2002). The figure shows the application of this mechanism to a ring with *n* = 10. The stochastic time traces correspond to the protein expression of proteins *p*_*j*_ with *j* = 1, 3, 5, 7, 9 and the corresponding control (top) in response to proteins *p*_1_ and *p*_2_ (trace not shown). The right figure is a magnification of the dashed square inside the main figure. We have also checked that this control protocol is applicable for rings with as low as *n* = 6 genes (not shown).

**Table 1.** Bifurcation analysis and unstable periodic solutions of repressilator rings with even number of genes. We use the continuation package Auto (Doedel 2007) to obtain the bifurcations of rings of size *n* (1). The parameter *c*, defined in equation (2.2), is swept in the biologically relevant range *c* ∈ [0.001,30] by changing *c*_1_ with *c*_2_ = 0.12, *c*_3_ = 0.16 and *c*_4_ = 0.06 constant. In agreement with analytical calculations, a branching point corresponding to a pitchfork bifurcation (P) is found at *c* = 2. A series of HBs linked to the emergence of unstable periodic solutions are found subsequently. Floquet analysis indicates that the first unstable orbit to emerge has only one unstable direction, regardless of the dimension of the system, and that the maximal Floquet multiplier decreases with increasing *n*. Hence, this periodic solution is quasi-stable: if it is reached, the divergence away from it is slow, and gets slower for longer rings. Other unstable orbits are present but their high instability makes them irrelevant to the observed dynamics. A similar structure of unstable orbits exists in odd rings (see the electronic supplementary material). The figure on the right shows the bifurcation diagrams for even rings of length *n* = 6, 12, 16. The unstable periodic orbits, shown as dark grey dashed lines, emerge through HBs.

*n*	bifurcation	*c*	stable/all directions	max. Floquet	period (min)	
2	P	2.00	—	—	—	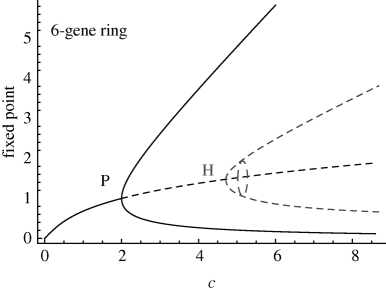
4	P	2.00	—	—	—	
6	P	2.00	—	—	—	
	HB	4.68	11/12	6.2	132	
8	P	2.00	—	—	—	
	HB	3.00	15/16	3.9	186	
10	P	2.00	—	—	—	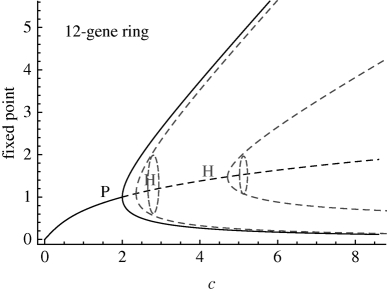
	HB	2.56	19/20	3.0	239	
	HB	9.62	17/20	9.0	104	
12	P	2.00	—	—	—	
	HB	2.36	23/24	2.5	291	
	HB	4.68	21/24	6.2	132	
14	P	2.00	—	—	—	
	HB	2.24	27/28	2.2	342	
	HB	3.51	25/28	4.8	160	
	HB	16.9	23/28	10.5	94	
16	P	2.00	—	—	—	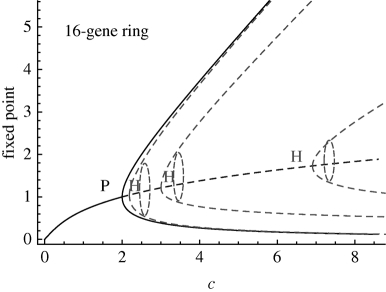
	HB	2.18	31/32	2.0	393	
	HB	3.00	29/32	3.9	186	
	HB	6.91	27/32	7.8	113	
18	P	2.00	—	—	—	
	HB	2.15	35/36	1.8	444	
	HB	2.71	33/36	3.4	213	
	HB	4.71	31/36	6.2	132	
	HB	27.33	29/36	11.5	89	

The existence of quasi-stable modes provides us with the opportunity to design a distinct control strategy. If we operate the system to revolve around a quasi-stable mode, the result is an oscillator that can be switched on, kept oscillating and switched off on demand. Below, we introduce a simple implementation of such a scenario and evaluate its robustness of operation. Note that an intricate family of unstable periodic orbits with high symmetry exists both in odd and even rings (table 1 and electronic supplementary material). However, these additional periodic solutions have several unstable directions that make them essentially unobservable and uncontrollable.

### 4.2. Spatio-temporal structure of the periodic solutions

The spatio-temporal structure of the periodic solutions, both in the odd and even cases, corresponds to a travelling-wave solution propagating around the ring. The snapshots in figure 2*b*,*c* show that this propagation occurs against the backdrop of the dimerized fixed point solution of the even ring, where all odd (even) numbered genes are ‘up’ while the even (odd) numbered genes are ‘down’ (Elowitz & Leibler 2000). Clearly, a dimerized configuration cannot be accommodated in an odd ring. This leads to a kink-like (frustrated) solution, where two consecutive genes have similar expression levels. This local imbalance of repression induces a dynamical instability that makes the kink propagate around the odd ring in a periodic fashion (figure 2*b*). This spatio-temporal structure underlies the limit cycle solution in odd rings. The fact that the period of the limit cycle increases (roughly) linearly with the number of genes indicates that the speed of propagation of the kink is (roughly) constant.

The quasi-stable periodic solution in even rings can be interpreted under the same prism. Figure 2*c* shows that it corresponds to *two interacting kinks* propagating around the ring at a roughly constant speed with a period that is approximately one-half of that of the closest odd ring (figure 2*a*). The instability of this periodic solution has a clear meaning in this picture: if the two kinks ‘collide’, they annihilate each other and the system returns to the stable fixed point, i.e. the dimerized solution. Figure 2*c* also shows that each kink has a minimum spatial width that depends on the parameters of the model. Hence, it is more difficult to find these oscillatory solutions in rings that are not large enough to fit two such perturbations although they can still be observed in smaller, biologically realizable rings (see the electronic supplementary material). For clarity, we have chosen to illustrate the spatio-temporal structure of the solutions with long rings. However, we have checked that the quasi-stable periodic orbits in rings with *n* = 6, 8, 10 (not shown) maintain the features of the two-kink structure and operate under the same principles as the long rings shown in figure 2*c*.

The spatio-temporal structure of the periodic solutions in repressilator rings shows a strong parallelism with similar dynamical solutions observed in classical models of discrete lattices (Braun & Kivshar 2004). The travelling-wave nature of the oscillations could have potential biological applicability if one were to use this circuit as a control element for genes that must be activated in a particular order and for a predefined time interval.

### 4.3. Robust induction of quasi-stable oscillations in the deterministic regime

To test that the oscillations described above are reachable, and therefore observable, we have carried out a sampling of the convergence to such solutions from random initial conditions. The results are presented in figure 3*a*,*b*, where we show that the proportion of initial conditions leading to sustained oscillations in even rings increases as the ring becomes longer but are already substantial for *n* = 10. This indicates that such oscillations could be observable in transient dynamics, especially in the presence of noise. This is highlighted by stochastic numerics (figure 3*b*, inset) that show an increased observability of these oscillations in the corresponding stochastic system.

The causality imposed by the travelling wave structure means that the manipulation of one gene will have a predictable effect on the others. This means that we can use such causality to devise a protocol to induce and stop oscillations in even rings reliably by activation of one gene for a short time span. We illustrate this simple scenario in figure 3*c*. First, the even ring is forced to converge to one of the fixed point solutions with a STOP signal that consists of the external activation of gene *j* for a time interval longer than the period of the oscillation. This signal is used to ‘initialize’ the system, suppressing any transient oscillations present in the system. Once the system is at rest, the oscillation can be started with a KICK signal, consisting of the external activation of gene *j* + 1 with a step function of width and amplitude similar to those of the oscillatory pattern. Such signals can be elicited non-invasively through an optical mechanism that uses UV or red light to activate the production of mRNAs of particular genes (Shimizu-Sato *et al.* 2002; Levskaya *et al.* 2005).

The STOP–KICK scenario induces long-lived oscillations with absolute reliability in rings with *n* > 6 identical genes. In order to check that the proposed protocol is robust to parameter variations, we have carried out a global robustness analysis as outlined in §3. We construct a hypercube by taking variations of 5, 10 and 20 per cent around the reference values of the parameters in equation (2.1) and take 10^4^ samples in this hypercube varying all parameters simultaneously. Sampling is performed with quasi-random reverse Halton sequences for improved convergence (Halton 1960; Vandewoestyne & Cools 2006). Figure 3*d* shows that the fraction of parameter samples that lead to oscillations with this protocol converges to 1 for large rings. Our numerics also show that oscillations can be elicited with significant robustness in rings with *n* > 6. When oscillations are present, the period shows very small variation with respect to the reference set, as shown by the coefficient of variation (figure 3*d*, inset). We have also quantified the change in the shape of oscillations through a normalized mean square measure and found that the shapes in the perturbed system exhibit very high similarity (approx. 99%) to the reference set (see the electronic supplementary material). In summary, our global robustness analysis indicates that in the deterministic regime, long-lived quasi-stable periodic solutions are reliably inducible in larger rings with a single intervention. For moderate size rings, oscillations can still be induced for a large fraction of the parameter hypercube but are short-lived. This suggests that repeated interventions could be used in order to keep the ring in the quasi-stable oscillating state. A simple control protocol that implements these ideas is proposed in the following section and shown to be applicable for rings as small as *n* = 6 operating in the stochastic regime.

### 4.4. Stochastic oscillations in even rings and readout-based control

We have used the standard Gillespie algorithm (Gillespie 1977) to study the generalized repressilator in the stochastic regime, i.e. when intrinsic noise is high owing to low copy numbers. It is well known that stochastic models of odd rings behave as oscillators and that the travelling wave structure is preserved (Elowitz & Leibler 2000; Hemberg & Barahona 2007). In the case of even rings, we have performed long stochastic simulations (not shown) and found bistability and switching events, as expected from the long-term attractors of the underlying deterministic system. Additionally, the simulations show sustained oscillatory behaviour, especially in longer rings although they are also observable in rings as small as *n* = 6 (see the electronic supplementary material). The oscillations appear in a variety of settings: as transients from a variety of initial conditions; spontaneously emerging from one of the stable points; or associated with switching events. It is also easy to induce such oscillations with localized interventions in particular genes; to sustain them with periodic driving; and to terminate them with a prolonged induction of a gene (as in the STOP signal above). We have examined the structure of these oscillations and they correspond well with the quasi-stable periodic solutions in the deterministic system: their period is approximately half the period of the closest odd ring (figure 2*a*) and the spatio-temporal travelling wave structure is maintained.

Our numerical simulations confirm the relevance of the underlying quasi-stable oscillations for the observed stochastic dynamics of even rings (figure 3*b*, inset). Similar to the deterministic case, the quasi-stable mode can also be used as a control operating point such that the system becomes switchable. The robust reachability of this mode allows us to use an extremely simplified feedback mechanism that could be implemented through an optical readout (GFP, YFP or luciferase protein labelling) and response (on-demand UV or red light gene transcription activation; Shimizu-sato *et al.* 2002). The simple control scheme illustrated in figure 4*a* uses the optical readout from two successive proteins in the ring to introduce optical KICK signals that sustain the oscillation based on a threshold rule (figure 4*b*). The oscillation can be started and terminated using the same optical signals. Although we have chosen to illustrate the possible implementation of the scheme with light sensitive inducers, it is worth remarking that any suitable mechanism capable of precisely timed gene induction with good spatial resolution at the cell population could be used. A potential advantage of a switchable mode of operation is the economical and targeted use of the transcriptional resources without overburdening the cell with unnecessary mRNA production (A. Glieder 2009, personal communication).

## 5. Discussion

In this work, we have studied how the presence of quasi-stable periodic solutions affects the observable dynamics of even repressilator rings. Previously, even rings have been thought of as switches owing to the fact that they only support fixed point solutions. However, our bifurcation analysis reveals the existence of a set of unstable orbits, some of which have slow time scales associated with them. These quasi-stable periodic solutions are both reachable and long-lived, thus playing a role in the observed dynamics, both transient and stochastic. This suggests that oscillatory behaviour might be more widespread than expected in genetic models, since it could feature in systems that possess only static attractors.

The presence of quasi-stable solutions provides us with the possibility of designing control protocols that operate the system around such modes, so that the oscillations can be turned on and off reliably. Our numerics indicate that a robust mechanism could be implemented based on appropriate optical feedback to switch the system between stable fixed points and quasi-stable oscillations. Although the proposed pared-down control scheme is only intended to provide an illustration of the potential implementation and its performance could be improved using optimized strategies for stochastic and robust control that take into account specific details of the experimental setup, some of its limitations are worth discussing. The challenge for the dynamical control algorithm is to deliver the optical interference signal necessary for the induction of gene expression for a short time period, to a particular spatial area of the cell population, and with a well-controlled delay following the fluorescent signal of the proteins. The proposed scheme shows both enough spatial and time resolution to address individual cells in a population with well-defined pulses (Shimizu-Sato *et al.* 2002). The scheme would need to rely on proper calibration of lifetimes of fluorescent proteins affected by phototoxic and photo-bleaching effects, as reviewed in detail by Bennett & Hasty (2009). Finally, we note that the delay between the actual protein concentration and the corresponding fluorescent signal introduced by the maturation time (approx. 2–8 min) is short when compared with the period of the oscillation (approx. 130 min), hence acceptable for the control scheme.

A synthetic circuit operating under such principles could be interfaced with a naturally occurring network to induce an intrinsic interference that is interruptible on demand. The switchability of this regulatory element can help avoid the appearance of adverse cumulative effects. The NFκB pathway is an example where such a regulator could provide controlled activation over short time intervals as an alternative to conventional knock-downs and other functional interventions that modify the balance of important proteins for the cell cycle (Karin & Lin 2002; Naugler & Karin 2008). The underlying travelling wave structure of the observed periodic solutions could also be potentially useful for design purposes. It allows for coordinated intervention when the timing and order of activation of different pathways is crucial. Examples of cellular networks, e.g. in developmental biology, indicate that timed patterns of sequential activation are at the heart of the function of families of master regulators (Holtzendorff *et al.* 2004; Liu *et al.* 2007; Ashall *et al.* 2009; Spencer *et al.* 2009) and, in the case of the vertebrate segmentation clock (Yun-Jin *et al.* 2000; Pourquie 2003), the associated oscillations are well defined but do not survive in the long term. The importance of heterogeneously timed gene induction has also been highlighted in a model of arabinose uptake in *E. coli* (Megerle *et al.* 2008). Experiments with genetically engineered yeast have also shown that pulsed activation of chaperons followed by pulsed activation of the associated heterologous proteins is more efficient at maximizing the production of particular metabolites (A. Glieder 2009, personal communication). These applications hint at potential uses for circuits that can produce sequential patterns of activation on demand, such as the even repressilator studied here, which interact with other cellular pathways via intrinsic proteins, thus avoiding the timed delivery of external agents through the cell membrane.

The design of control strategies for the operation of systems around an inherently unstable state has a long history in other disciplines (e.g. flight and fluid control) since it affords enhanced responsiveness and controllability with relative ease and simplicity of design (Franklin *et al.* 1993). This strategy differs fundamentally from the biochemical alteration of the network topology proposed by Atkinson *et al.* (2003) based on a smaller gene circuit but with a complex regulatory scheme involving promoter and repressor sites regulating one gene. The molecular kinetics of such regulators are less well understood than those with single regulatory sites owing to unavoidable cross-talk and compound logic. The ring topology relies on simple regulation to provide a sequence of causal signals but at the expense of involving a larger number of genes.

The present scheme is also in contrast with previously engineered gene circuits, such as odd repressilators, which possess globally attracting limit cycles leading to behaviour that is robust yet not controllable. Quasi-stable operation, on the other hand, is robustly switchable. The switchability of the oscillator coupled with dynamic control that affords good spatial resolution could be used to elicit localized oscillations in cell populations as an aid to examine mechanisms of cell synchronization. It is an open area of current research to elucidate the role of a design concept based on control around unstable behaviour, similar to the inverted pendulum in classic control theory, to further our understanding of cell strategies and its potential use in the design of synthetic topologies that can interfere with naturally occurring pathways.
